# Genome-wide screening of the *RNase T2* gene family and functional analyses in jujube (*Ziziphus jujuba* Mill.)

**DOI:** 10.1186/s12864-023-09165-z

**Published:** 2023-02-20

**Authors:** Zhi Luo, Yu Zhang, Chunjiao Tian, Lihu Wang, Xuan Zhao, Zhiguo Liu, Lili Wang, Lixin Wang, Jin Zhao, Jiurui Wang, Mengjun Liu

**Affiliations:** 1 College of Horticulture, Hebei Agricultural University, Baoding, 071001 China; 2 Research Center of Chinese Jujube, College of Horticulture, Hebei Agricultural University, Baoding, 071001 China; 3 College of Forestry, Hebei Agricultural University, Baoding, 071001 China; 4 School of Landscape and Ecological Engineering, Hebei University of Engineering, Handan, 056038 China; 5 College of Life Science, Hebei Agricultural University, Baoding, 071001 China

**Keywords:** *Ziziphus jujuba* Mill., *RNase T2*, *ZjRNase1*, *ZjRNase2*, Seed

## Abstract

**Background:**

Ribonuclease (*RNase T2*) plays crucial roles in plant evolution and breeding. However, there have been few studies on the *RNase T2* gene family in *Ziziphus jujuba* Mill., one of important dried fruit tree species. Recently, the released sequences of the reference genome of jujube provide a good chance to perform genome-wide identification and characterization of *ZjRNase* gene family in the jujube.

**Results:**

In this study, we identified four members of *RNase T2* in jujube distributed on three chromosomes and unassembled chromosomes. They all contained two conserved sites (CASI and CASII). Analysis of the phylogenetic relationships revealed that the *RNase T2* genes in jujube could be divided into two groups: *ZjRNase1* and *ZjRNase2* belonged to class I, while *ZjRNase3* and *ZjRNase4* belonged to class II. Only ZjRNase1 and ZjRNase2 expression were shown by the jujube fruit transcriptome analysis. So *ZjRNase1* and *ZjRNase2* were selected functional verification by overexpression transformation of *Arabidopsis.* The overexpression of these two genes led to an approximately 50% reduction in seed number, which deserve further attention. Moreover, the leaves of the *ZjRNase1* overexpression transgenic lines were curled and twisted. Overexpression of *ZjRNase2* resulted in shortened and crisp siliques and the production of trichomes, and no seeds were produced.

**Conclusion:**

In summary, these findings will provide new insights into the molecular mechanisms of low number of hybrid seeds in jujube and a reference for the future molecular breeding of jujube.

**Supplementary Information:**

The online version contains supplementary material available at 10.1186/s12864-023-09165-z.

## Background

RNA depolymerase, also known as ribonuclease (*RNase*), is a type of acidic endonuclease that belongs to the *RNase T2* family [1]. In 1959, *RNase T2* was identified in *Aspergillus* fungi [2]. Recent studies showed that S-glycoproteins in tobacco are responsible for self-incompatibility and are highly homologous to *RNase T2* and *RNase Rh* from *Rhizopus niveus* [3–7]. S-glycoproteins have *RNase* activity and *RNase T2* family members have two conserved active-site fragments (CASI and II) [7]. These findings indicated that the function of RNase T2 family members is closely related to plant gametophytic self-incompatibility, and the *RNase T2* genes have been highly conserved throughout evolution. These discoveries could promote researches on the mechanism of plant self-incompatibility.

An increasing number of *RNase T2/S-RNase* enzymes have subsequently been discovered in the genomes of plants in Acacia, Solanaceae and Rosaceae [3, 8–10]. Among them, *RNases* were found in Japanese pear (*Pyrus*) and were shown to be involved in self-incompatibility of gametophytes [11]. The *S-RNase* genes of apricot (*Prunus armeniaca*) and loquat (*Eriobotrya japonica*) have also been identified as being related to gametophytic self-incompatibility [12, 13]. Thus, the *RNase T2/S-RNase* enzymes are closely related to gametophytic self-incompatibility and should be widely studied to increase the efficiency of hybrid breeding. Furthermore, it has been found that *RNases* is involved in abiotic stress responses such as salt stress, phosphate starvation and senescence [14].

Jujube (*Ziziphus jujuba* Mill.) a deciduous fruit tree species native to China, has been distributed worldwide [15–18]. Low fruiting set and seed production were key obstacles for hybrid creation in jujube cross breeding and no hybrid cultivar was successfully utilized in cultivation [18]. The exploration of *RNase T2* family members and functions will be helpful for jujube cross breeding. However, the work on jujube RNase family has yet not been conducted, and the functions of ZjRNase are still unclear. The aim of this study is to identify the RNase T2 gene family in jujube genome and demonstrate the potential function of RNase T2 genes. The results provided new insights into the molecular mechanisms of low number of hybrid seeds in jujube and found new functions of *RNase T2* family members in leaf and fruit development.

## Results

### Genome-wide identification of RNase T2 family members

Eight *RNases* have been identified in *Oryza sativa* [1]. The identification of jujube *RNase T*2 was performed via BLASTP searches and the *Oryza sativa* RNases protein sequences were used as query sequences to search the *Ziziphus jujuba* genome [19]. A total of 4 *ZjRNases* were identified by two rounds of BLASTP and conserved domain predictions (Table 1). The protein length from ZjRNase1 to ZjRNase4 is 226,280,242 and 158 and their encoding genes are located on chromosomes Chr1, Chr9, Chr10 and ChrUn, respectively. The predicted pIs ranges from 5.12 to 7.81, and the molecular weight is between 18.61 and 31.08 kDa. The instability index analysis showed that ZjRNase1, ZjRNase3 and ZjRNase4 are stable, except for ZjRNase2.The higher the aliphatic index was, the more stable the protein: among the jujube *RNase T*2 family members. *ZjRNase3* is the most stable one, followed by *ZjRNase4*, *ZjRNase2* and *ZjRNase1*. The *ZjRNase* grand average of hydrophobicity is negative, indicating that the proteins were slightly hydrophobic.

**Table 1:** Putative *RNases* in *Ziziphus jujuba*

Gene	Protein length (aa)	Chromosome location	Mol. Wt.(kDa)	pI	GRAVY	Instability index	Aliphatic index
*ZjRNase1*	226	Chr10	25.78	5.12	−0.348	37.50	71.64
*ZjRNase2*	280	Chr9	31.08	5.68	−0.060	50.33	77.00
*ZjRNase3*	242	Chr1	27.18	6.43	−0.183	39.22	91.40
*ZjRNase4*	158	ChrUn	18.61	7.81	−0.583	36.74	80.19

### Phylogenetic analysis and multiple sequence alignment of ZjRNases

**Fig. 1:**
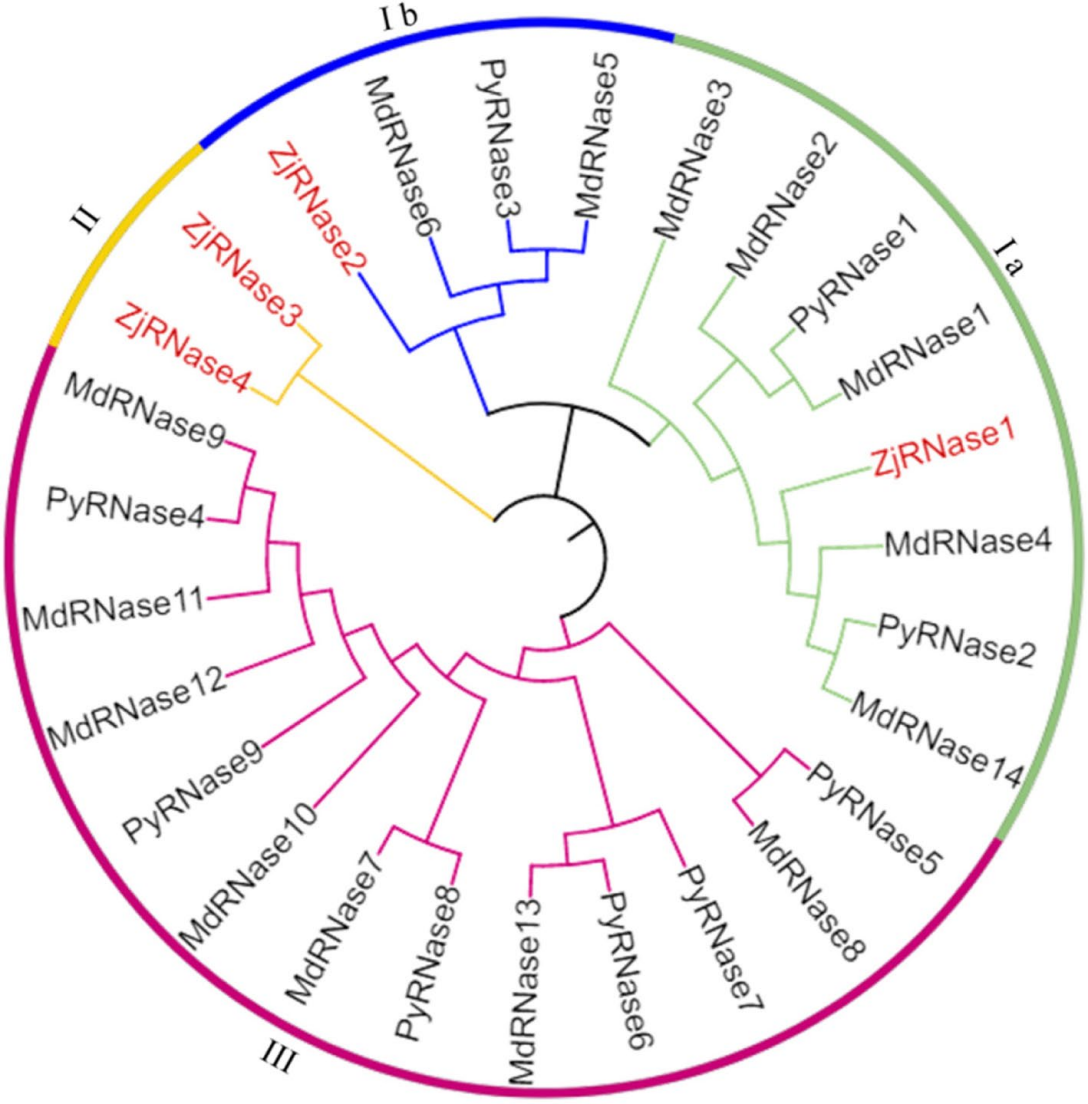
Rooted phylogenetic tree representing relationships between RNase proteins of *Pyrus, Malus domestica* and *Ziziphus jujuba.* All the RNase proteins were divided into three classes, class I was divided into two subgroups, which were represented by different coloured clusters. The phylogenetic tree was constructed by the NJ method using MEGA 7 software with 1000 bootstrap replicates

To better determine their evolutionary relationship and facilitate the classification of ZjRNases, a phylogenetic tree was constructed comprising the sequences of 14 MdRNases, 9 PyRNases and 4 ZjRNases (Fig. 1). The 4 ZjRNases were divided into two types: class I and class II (Fig. 1). ZjRNase1and ZjRNase2belongs to class I subgroups I a and I b, ZjRNase3 and ZjRNase4 belong to class II.

**Fig. 2:**
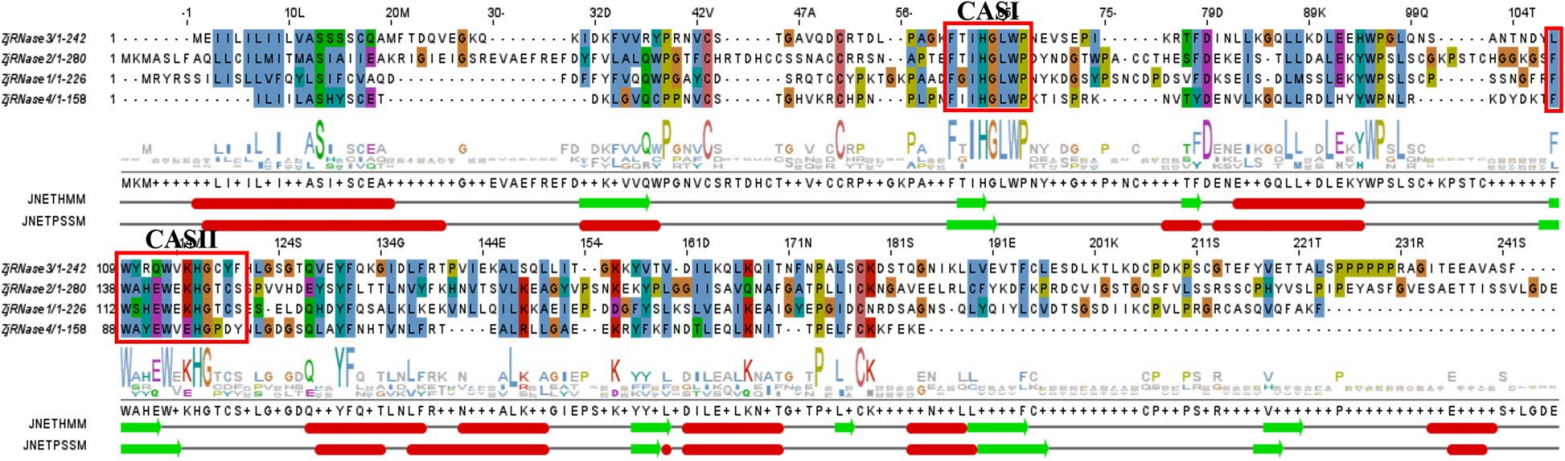
Multiple sequence alignment of the conserved active sitesof *ZjRNases*. The alignment was constructed by MUSCLE and visualized by Jalview. The two conserved active site (CAS red thick box) regions were indicated. The protein secondary structures were predicted using HMM and PSSM software. The red boxes represent α-helices, and the light green boxes represent β-sheet

Previously reported RNases have two conserved active sites, CASI and CASII motifs [20]. All the ZjRNases have the same conserved structural sites as the RNases in other plant species, suggesting that the four ZjRNase members we obtained in jujube are correct (Fig. 2). According to secondary structure analysis, it was predicted that α-helix or β-sheet structures are present at 14 positions, of which 7 (50%) may be α-helix structures (red) and 5 (35.71%) may be β-sheet structures (light green). The remaining two predictions were inconsistent and were classified as uncertain types. These results showed that the secondary structure of the ZjRNase member proteins are relatively stable.

### Analysis of the structural and conserved motifs of ZjRNases

**Fig. 3:**
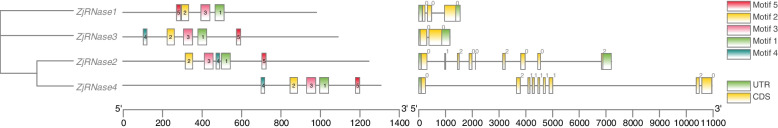
Phylogenetic relationships, gene structure and gene architecture of the conserved protein motifs in ZjRNases. **a** Phylogenetic tree was constructed based on the full-length sequences of ZjRNase proteins. **b** Motif composition. The motifs, numbered 1–5, were displayed in different coloured boxes. **c** Exon–intron structure of *ZjRNase*s. The light green boxes indicate untranslated 5′- and 3′-regions; the yellow boxes indicate exons; and the black lines indicate introns

The results of further analysis of the gene structure and motifs of the *ZjRNase*s were shown in Fig. 3. The phylogenetic tree confirmed that ZjRNases could be grouped into two classes (Fig. 3a). Analysis of the genomic DNA sequences showed that *ZjRNase*s usually have 1, 2 or more than 2 introns (Fig. 3c). *ZjRNase1*has two introns, while *ZjRNase3* has one intron. *ZjRNase2/4* has more than 2 introns (Fig. 3c). MEME analysis was performed online to identify additional motifs among the 4 *ZjRNases*. Five conserved motifs were predicted (Fig. 3b), and each *ZjRNase* contained four or five of them. Several motifs were common to most members. Compared to the other three members, *ZjRNase1* lacked motif 4 (light blue box in Fig. 3b).

### Analysis of cis-acting elements in the promoter region of ZjRNases

**Fig. 4:**
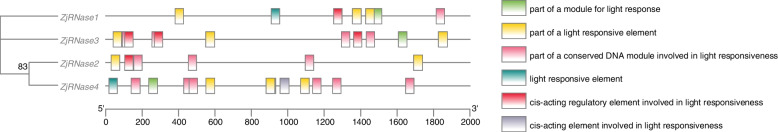
Prediction of cis-acting elements in *ZjRNases*. a Numbers of cis-acting elements detected in the promoter region of each *ZjRNase* gene. All the cis-acting elements were divided into six types

The cis-acting elements were found to be related to hormone responses, development, light responses, promoter site binding and other functions (Fig. 4). The important elements are light-responsive elements, including Box 4, G-Box, TCT motif, CAAT-box, GATA motif and GATA motif elements. Six hormone-responsive elements were identified, and the jujube T2 family members were found to contain 6 to 12 light-responsive elements.

### Analysis of protein structure and protein interactions

**Fig. 5:**
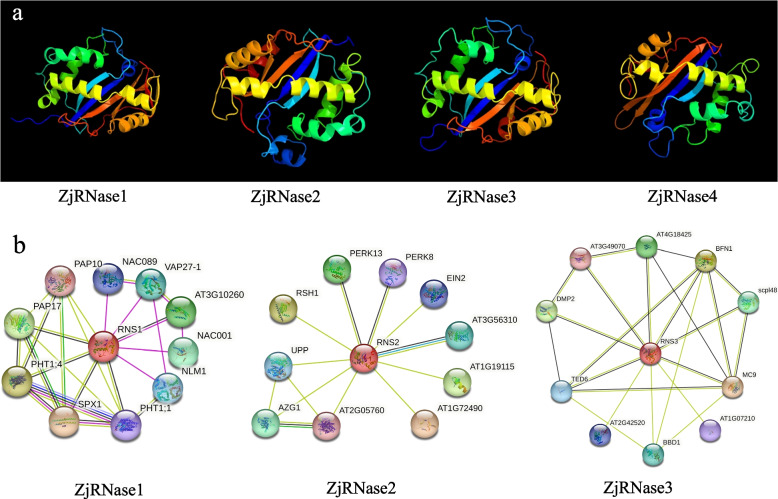
ZjRNase gene family protein analysis. **a** Protein structure analysis of 4 ZjRNases via the Phyre2 database. **b** Protein–protein interaction analysis of 3 ZjRNases by the STRING database

The results of protein structure prediction analysis showed that ZjRNase1 is similar to ZjRNase3 and that ZjRNase2 is similar to ZjRNase4. These results are consistent with those of the above-mentioned gene structural analysis (Fig. 5a). Protein interaction prediction analysis predicted only AtRNS1 (ZjRNase1), AtRNS2 (ZjRNase2), and AtRNS3 (ZjRNase3). AtRNS4 (ZjRNase4) had no prediction results (Fig. 5b). Many proteins interacted with *ZjRNase* proteins, some of which were transcription factors, such as NAC (*NAC* genes) and EIN2 (INSENSITIVE 2).

### The transcriptome sequencing analysis of jujube fruits

**Fig. 6:**
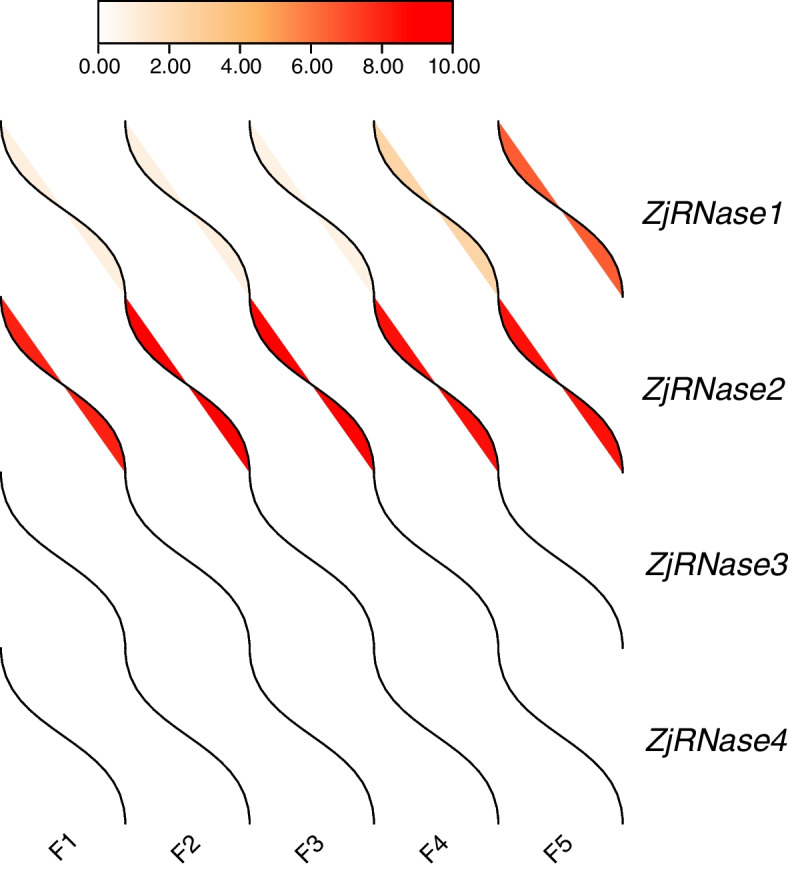
The leaves morphology of the *ZjRNase1* three representative transgenic lines. F1 represents the young fruit period, F2 represents the bulge period fruit, F3 represents the white-ripe period fruit, F4 represents the half-red period fruit, and F5 represents the whole-red period fruit

Further through the analysis of the published winter jujube transcriptome data showed that *ZjRNase1* and *ZjRNase2* were expressed in jujube fruits, but hardly *ZjRNase3* and *ZjRNase4* (Fig. 6)*.* The results indicate that *ZjRNase1* and *ZjRNase2* may be involved in fruit and seed development. In order to further understand the function of *ZjRNase1* and *ZjRNase2*, subsequent transgenic experiments were carried out.

### Overexpression of ZjRNase1 and ZjRNase2 in *Arabidopsis thaliana*

**Fig. 7:**
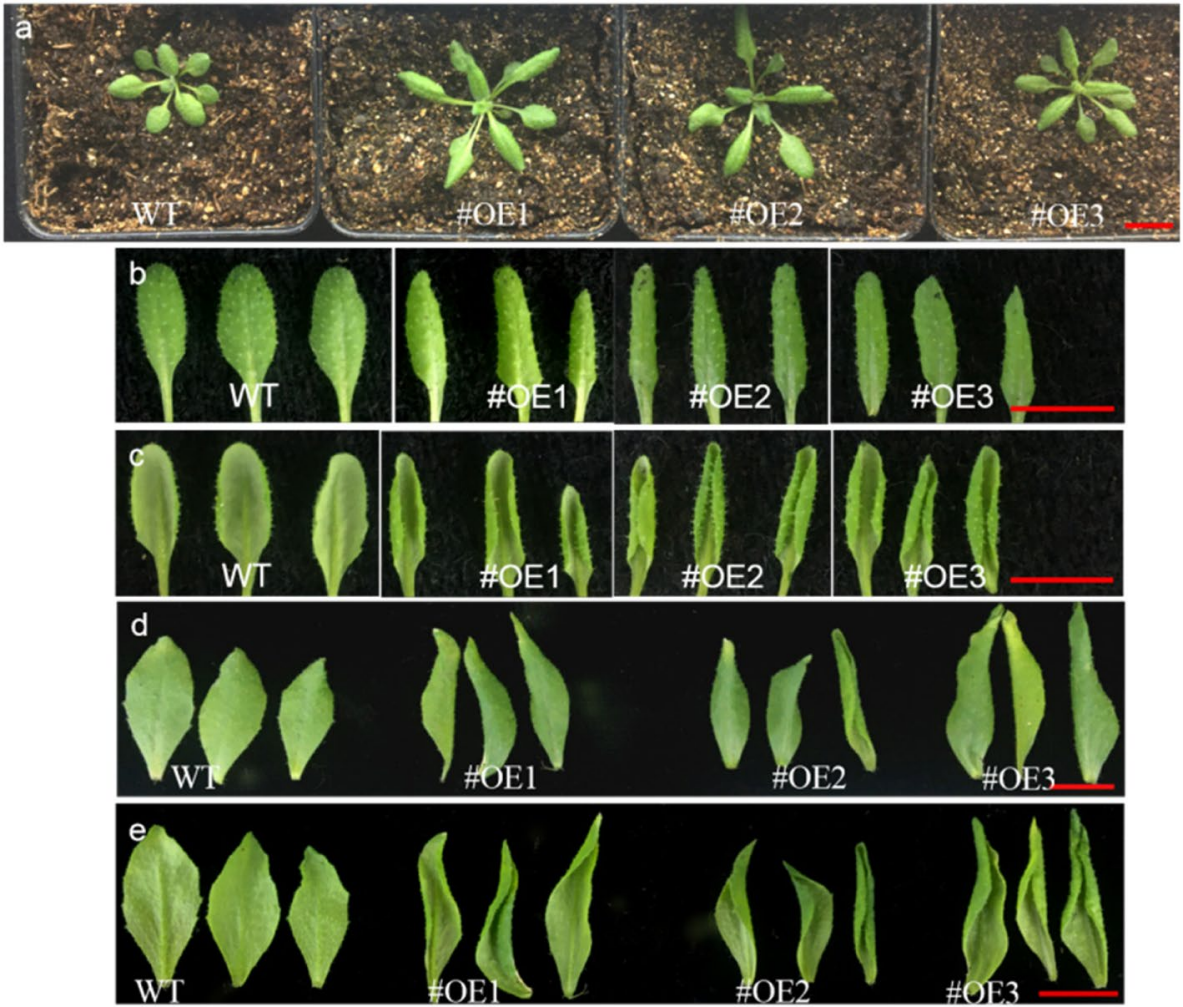
The leaves morphology of the *ZjRNase1* three representative transgenic lines (#OE1, #OE2 and #OE3) and WT. *Scale bar*.1 cm **a** Growth morphology of three representative *ZjRNase1* transgenic lines and WT after planted for 7 days. *Scale bar*.1 cm **b** The frontal morphology of rosette leaves of three representative *ZjRNase1* transgenic lines and WT after planted for 14 days. *Scale bar*.1 cm **c** The abaxial morphology of rosette leaves of three representative *ZjRNase1* transgenic lines and WT after planted for 14 days. *Scale bar*.1 cm **d** The frontal morphology of the inflorescence stem leaf of three representative *ZjRNase1* transgenic line and the WT after planted on the 21 days. *Scale bar*.1 cm **e** The abaxial morphology of the inflorescence stem leaf of three representative *ZjRNase1* transgenic line and the WT planted on the 21d. *Scale bar*.1 cm, WT: *Arabidopsis thaliana* (Col-0), OE: overexpression*ZjRNase1*

**Fig. 8:**
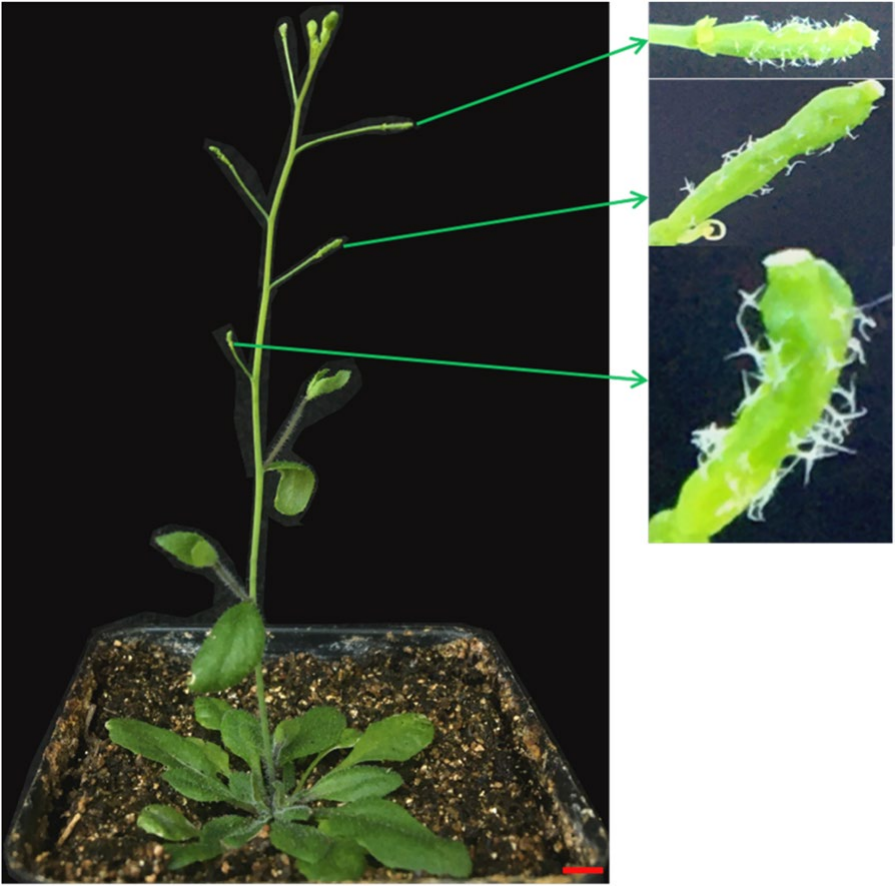
Morphology of siliques of transgenic *ZjRNase2* overexpression plants. *Scale bar*: 1 cm

Recombinant vectors overexpressing *ZjRNase1* and *ZjRNase2* were constructed. *Arabidopsis thaliana* (Col-0) plants were transformed via *Agrobacterium*-mediated infection, and the seeds were collected. Transgenic plants with *ZjRNase1* and *ZjRNase2* were obtained by screening resistant seedlings via hygromycin-containing media. The leaves of the transgenic lines overexpressing *ZjRNase1* were significantly curled and twisted (Fig. 7). Further observations under an anatomical microscope of the transgenic lines overexpressing *ZjRNase2* revealed that the siliques were crisp, with small trichomes around them (Fig. 8).

**Fig. 9:**
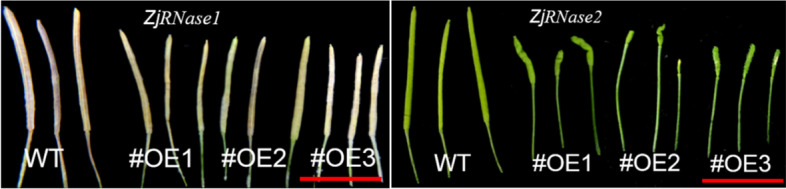
Morphology of siliques of the three representative transgenic *ZjRNase1*and *ZjRNase2*overexpression lines(#OE1,#OE2 and #OE3) and WT plants. *Scale bar*: 1 cm. WT: *Arabidopsis thaliana* (Col-0), OE: overexpression *ZjRNase1* and *ZjRNase2*

**Fig. 10:**
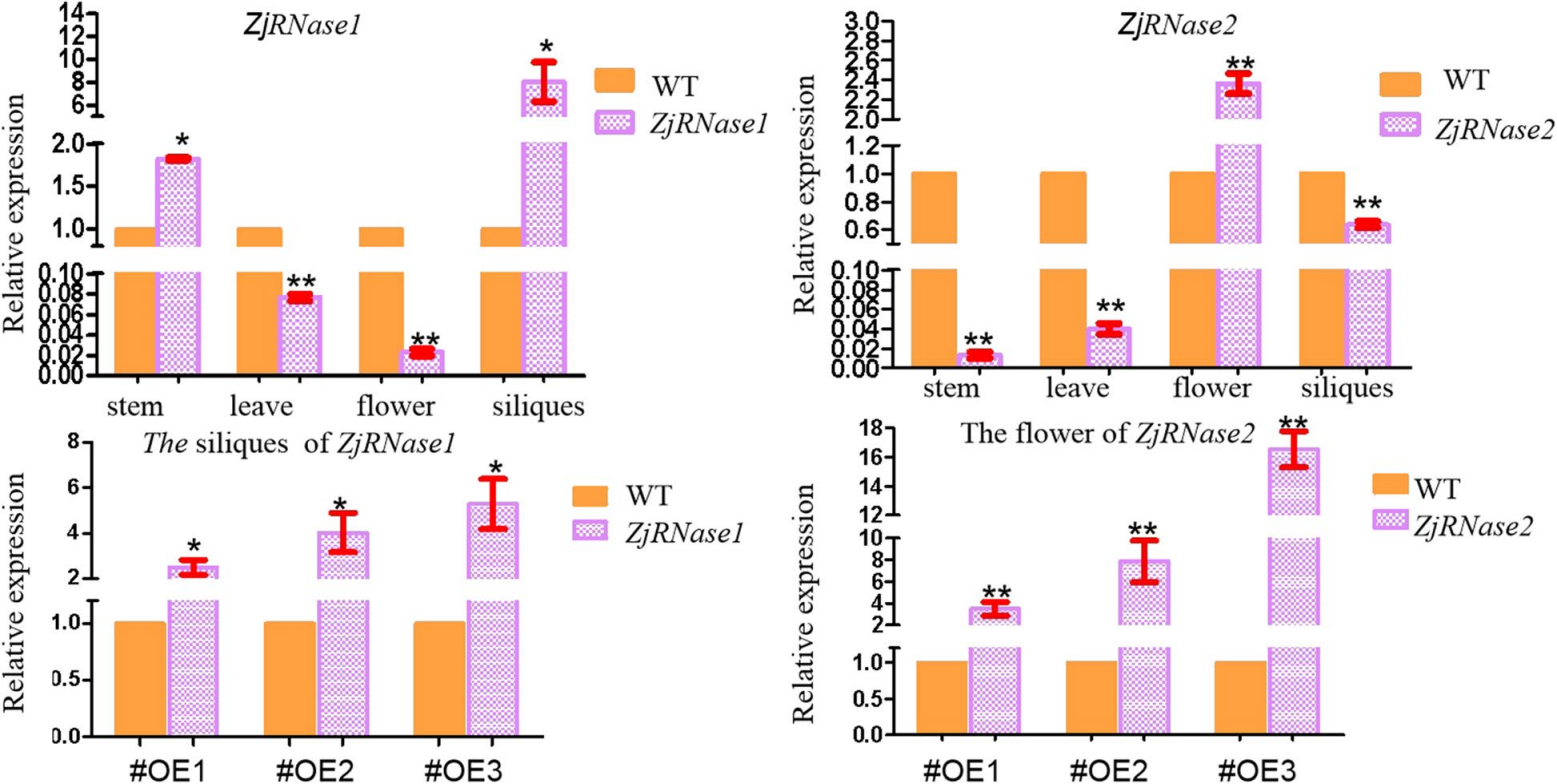
qRT-PCR detection of transgenic plants. a. Tissue-specific expression of the *ZjRNase1* and *ZjRNase2.* b. Tissue expression of the *ZjRNase1 ZjRNase2* three represenative transgenic lines (#OE1, #OE2 and #OE3) and WT. WT: *Arabidopsis thaliana* (Col-0), OE: overexpression *ZjRNase1* and *ZjRNase2*

The siliques of the *ZjRNase* overexpression plants were significantly shorter than those of the *Arabidopsis thaliana* (Col-0) plants (Fig. 9). Tissue-specific expression of the leaves, stems, flowers and siliques of the overexpression plants revealed that *ZjRNase1* was mainly expressed in the siliques, but *ZjRNase2* was mainly expressed in the flowers (Fig. 10).

The seeds were harvested from the T1 generation of *ZjRNase1* and *ZjRNase2* overexpression transgenic lines. The seeds were screened via media containing hygromycin, and the phenotype of the T3-generation overexpression plants was the same as that of the T2- and T1-generation plants.

**Fig. 11:**
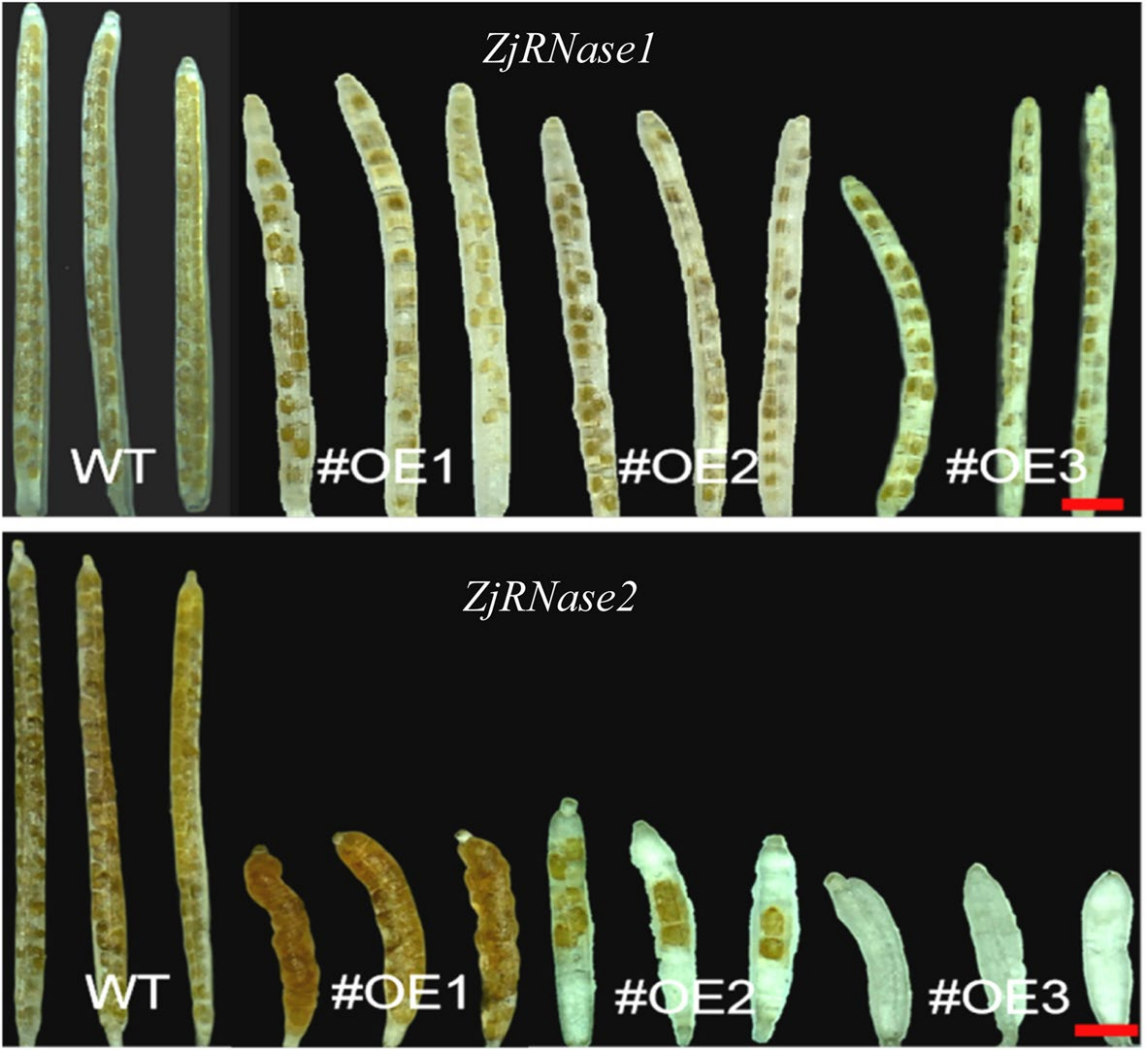
Seeds in the siliques of three representative transgenic lines (#OE1,#OE2 and #OE3) and WT. *Scale bar*: 1 mm, WT: *Arabidopsis thaliana* (Col-0), OE: overexpression*ZjRNase1*and *ZjRNase2*

**Fig. 12:**
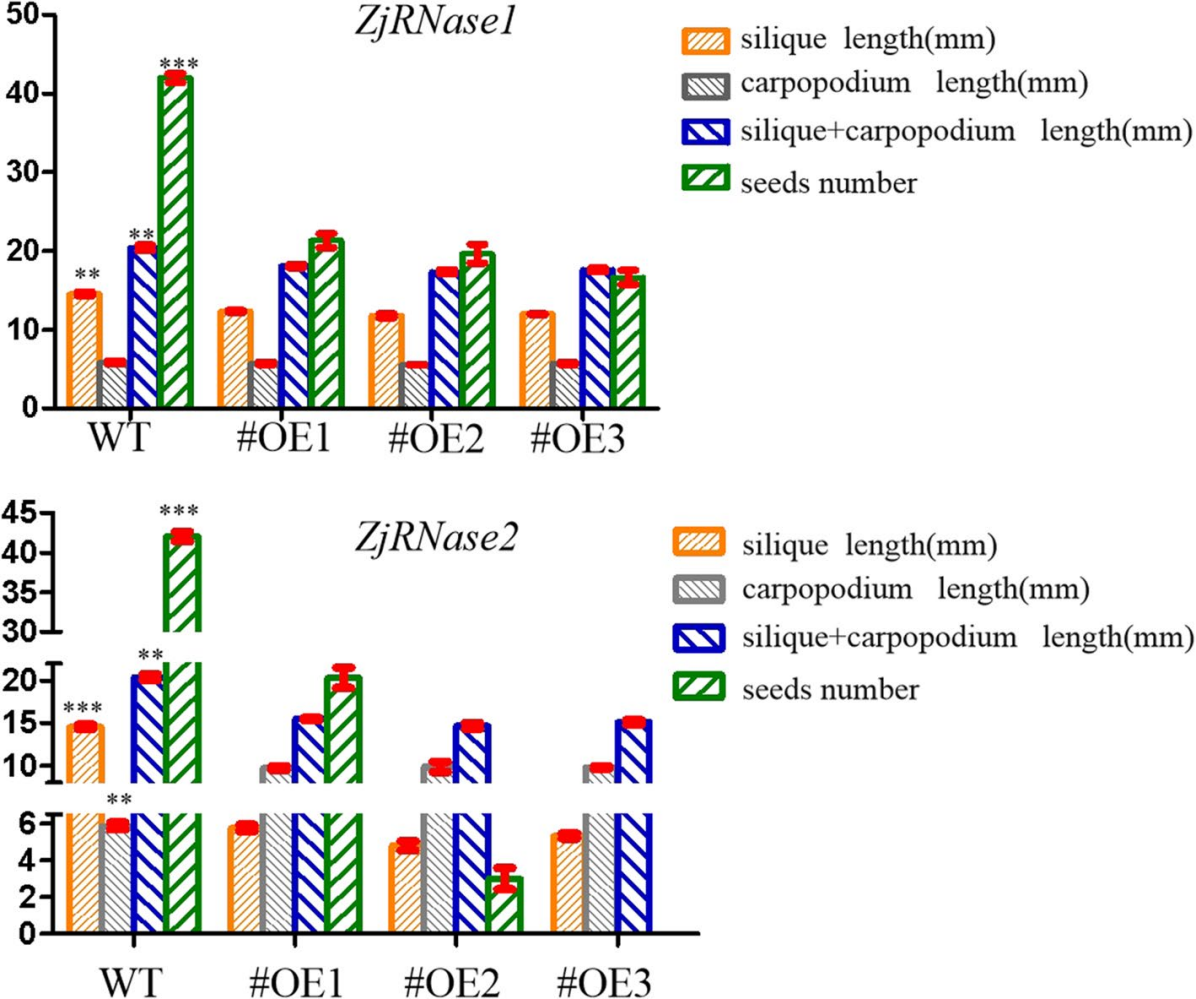
Statistical analysis of the siliques, carpopodium and seeds of three representative transgenic lines (#OE1,#OE2 and #OE3) and WT. The error bars represent the ±SDs of three independent replicates (***, *p* < 0.001;**, *p* < 0.01; *, *p* < 0.05 Duncan’s multiple range test).WT: *Arabidopsis thaliana* (Col-0), OE: overexpression*ZjRNase1*, *ZjRNase2*

Through statistical analysis of the siliques and seeds of the *ZjRNase1* and *ZjRNase2* overexpression plants, the siliques of the overexpression plants were significantly shorter than those of the *Arabidopsis thaliana* (Col-0), with the *ZjRNase2* overexpression plants presenting the most significant results, only 4.80 mm long (Fig. 11). In addition, the podetium length of the *ZjRNase2* overexpression plants was significantly longer than that of the *Arabidopsis thaliana* (Col-0). Compared with that of the *Arabidopsis thaliana* (Col-0), the number of seeds produced by the *ZjRNase1* and *ZjRNase2* overexpression plants was significantly reduced. The average seed number of the *ZjRNase1* overexpression plants was only approximately 50% that of the *Arabidopsis thaliana* (Col-0). Interestingly, the seed number significantly decreased for *ZjRNase2* overexpression lines 1, 2, and 3 to approximately 50, 8.33, and 0% of that for the *Arabidopsis thaliana* (Col-0), so seed production was absent in the *ZjRNase2* overexpression transgenic line 3 (Figs. 11 and 12). The results showed that overexpression of the *ZjRNase1* and *ZjRNase2* genes in *Arabidopsis* can cause reduced seed content. Additionally, this overexpression can cause morphological changes in other tissues. The leaves of the *ZjRNase1* overexpression transgenic lines were curled and twisted. Moreover, the siliques of the *ZjRNase2* overexpression lines were wrinkled with few or even no seeds.

## Discussion

Ribonuclease (*RNase T2*) play crucial roles in plant evolution and breeding and an increasing number of *RNase T2/S-RNase* enzymes have subsequently been reported in genome of different plants, such as Acacia, Solanaceae and Rosaceae [3, 8–10]. Through genome-wide mining, four members of the *RNase T2* family were identified in jujube. These members could be divided into two categories according to phylogenetic analysis, which is relatively low compared with that of other species [1, 5].

The previous study showed that *RNase T2* is related to self-incompatibility and abiotic stress responses in plants [1, 3, 5]. Some genes involved in seed and fruit development have been reported [21, 22]. For instance, *YABBY、OVATE and EPFL2 influenced fruit size and shape, ORANGE and MPK4* were related to seed number [23–29]. However, the research about *RNase T2* function during fruit and seed development was absent. In terms of bioinformatics, the *RNase T2* gene family members in jujube have characteristics similar to those of RNases of other plant species [1, 5]. In particular, the jujube *RNase T2* gene retains the original ability of T2 genes, and when overexpressed in *Arabidopsis*, these genes could lead to reduced seed production. The *VvNAC26* transgenic plants were found to reduce tomato seeds [30] and INSENSITIVE2 (EIN2) encodes a membrane protein and affected seed development [31]. This study showed that *ZjRNase1* and *ZjRNase2* had interaction with NAC and EIN2, respectively. It indicated NAC and EIN2 transcription factors were probably associated with function of *ZjRNase1* and *ZjRNase2* involved in seed development.

In addition, the jujube *RNase T2* family members showed some differences in terms of their function. The class I member *ZjRNase1*, after being overexpressed, was found to be highly expressed only in flowers and caused a twisted-leaf phenotype in addition to siliques shorten and reduced seed number. Analysis of its promoter revealed that the sequence (CAAT (A/T) ATTG) may participate in the differentiation of the palisade tissue and can also bind HD-ZIPI transcription factors, which have been shown to play a role in leaf morphogenesis [32]. In addition, HD-ZIPI proteins were identified as capable of interacting with RTNLB8 and VAP27-1 in terms of protein interactions [33]. All of these interacting proteins are highly expressed during leaf morphological development. The class II member *ZjRNase2* was expressed in the flowers of the transgenic lines. Overexpression of this gene caused crisp siliques, reduced seed numbers and even no seeds, as well as the production of trichomes. According to the literature, plants produce more trichomes to resist pests, indicating that such gene may also be associated with insect resistance [34, 35].

## Conclusions

In this study, four *ZjRNase* genes and their corresponding protein sequences were identified from the jujube genome, and the *ZjRNase* genes were divided into two categories. Class I had member *ZjRNase1* and *ZjRNase2, ZjRNase3* and *ZjRNase4* belonged to Class II but not expressed in the fruit. Therefore, *ZjRNase1* and *ZjRNase2* were selected for functional verification. As a result of the transgene, we founded that *ZjRNase1* and *ZjRNase2* could lead to a decrease in the number of seeds, inferring that *ZjRNase* genes might be involved in the formation of seeds. This study provides a basis for further studies on the functional properties of *RNase* genes; however, further studies are needed to better elucidate the regulatory mechanism of these four *RNase* genes on seed formation in jujube breeding.

## Materials and methods

### Plant materials and cultivation

*Arabidopsis thaliana* (Col-0) seeds were obtained from Xuan Zhao from China Agriculture University and sown in a soil medium matrix (peat: vermiculite = 1:1) under a 16 h light/8 h darkness photoperiod at 20 ± 2 °C and a relative humidity of 60 ± 5%. The seeds of *Arabidopsis thaliana* (Col-0) plants were grown on 1/2-strength Murashige and Skoog (MS) media. All the plants were grown under the same conditions. RNA was extracted using a TIANGEN plant RNAprep Pure Plant Kit DP432 (TIANGEN biotech company). Jujube fruit transcriptome data were obtained from the National Center for Biotechnology Information (NCBI) database [19].

### Database searches and identification of RNase genes in the *Ziziphus jujuba* genome

The genome sequences of *Ziziphus jujuba* Mill. were obtained from the National Center for Biotechnology Information (NCBI) database [19]. The sequences of 14, 9 identified *MdRNases*, *PyRNases* were downloaded from the National Center for Biotechnology Information (NCBI) database. In addition, the sequences of8 identified *OsRNases* were downloaded [1]. *RNases* were identified by two rounds of BLASTP searches. First, the sequences of all *OsRNases* were used to search for possible *ZjRNases* sequences via TBtools [36]. Then, NCBI Batch CD-Search was used to confirm whether the candidate *RNases* contained the *RNase_T2* superfamily domain (pfam00445) or the Ribonuclease_T2 domain (cl00208). A total of 4 *ZjRNase* genes were ultimately identified in the genome. The protein length, isoelectric point (pI) and molecular weight (MW) were subsequently predicted.

### Phylogenetic analysis and multiple sequence alignment

Sequences of the OsRNase proteins were obtained from the Phytozome database. A neighbour-joining (NJ) phylogenetic tree comprising the full-length sequences of MdRNases, PyRNases and ZjRNases was constructed with 1000 bootstrap replicates using MEGA 7.0. Multiple sequence alignment of all ZjRNases was also performed by MEGA 7.0.

### RNase gene structure and conserved motif analysis

The *RNase* gene structure and conserved domains were analysed and visualized using TBtools software [36]. The conserved motifs of the identified ZjRNase proteins were explored with the help of the Multiple Expectation Maximization for Motif Elicitation (MEME) online program.

### Analysis of cis-acting elements of ZjRNases

The potential regulatory cis-acting elements of jujube *RNases* were checked by using TBtools software; the region 2000 bp upstream of the start codon was evaluated. Then, the cis-acting elements in the promoter were predicted via PlantCARE online software to identify their regulatory functions.

### Protein structure and protein–protein interaction predictions

The amino acid sequences of 4 ZjRNases were submitted to Phyre2 (http://www.sbg.bio.ic.ac.uk/phyre2/html/) for protein structure analysis [37]. Similarly, the amino acid sequences of the same 4 ZjRNases were submitted to the STRING database (https://string-db.org/) for protein–protein interaction analysis. The orthologues of these genes in *Arabidopsis thaliana* were selected as references.

### RNA extraction and quantitative real-time PCR (qPCR) analysis

The extraction of total RNA from leaves and subsequent cDNA synthesis were performed as described previously [38]. Gene expression was then analyzed via qPCR [39]. We included at least three independent biological replicates and three technical replicates. First-strand cDNA was synthesized from RNA with a PrimeScript RT Reagent Kit (TIANGEN). qPCR was carried out on 20 μL reaction mixtures by the use of SYBR Green fluorescence (TransGen Biotech, China) in conjunction with a Roche LightCycler® 480 Real-Time PCR System [40]. The *AtActin2* was used as the internal reference control [39]. Relative gene expression levels were calculated according to the 2^-ΔΔCT^ method [41]. All the primers used for qPCR are listed in the Additional file 1.

### Plasmid construction and gene overexpression

According to the bioinformatics analysis above, *ZjRNase1* and *ZjRNase2* were selected for gene functional analysis. Plasmid construction and agroinfiltration assays of *Arabidopsis thaliana* (Col-0) were performed based on the sequences above. We cloned the coding DNA sequences (CDS) of *ZjRNase1* and *ZjRNase2*and inserted them into a stable overexpression vector (PBI121) [42]. All the primers used for qPCR are listed in the Additional file 1. The above-mentioned vector construct was infected into *Arabidopsis thaliana* (Col-0) through the transformation method mediated by Agrobacterium (GV3101 strain) and infection buffer (5% sucrose, 0.04% Silwet-77). Infected *Arabidopsis thaliana* (Col-0) plants was incubated in the dark for 24 h.

### Seed observation experiment

The ripe siliques were washed with distilled water, placed in a 95% ethanol and acetic acid (3:1) fixative solution for 24 hours, and then transferred to 70% ethanol and stored in a refrigerator at 4 °C overnight. The next day, it was transferred into 85 and 95% ethanol, dehydrated for 1 h respectively, and dehydrated in absolute ethanol 3 times, the first two times were 1 h, and the last time was 5 h. soak with ethanol and methyl salicylate (1:1) for 1 h, and soak with pure methyl salicylate for 3 times, the first 2 times for 1 h, and the last time for 24 h. Then observed under the microscope and took pictures for preservation.

### Statistical analysis

The experiments were performed for three technical replications. One-way analysis of variance (ANOVA) was used to determine the statistical significance at *p* ≤ 0.05.Statistically signifcant diferences were indicated either with * (*P* < 0.05), ** (*P* < 0.01), *** (*P* < 0.001).

## Supplementary Information

**Additional file 1.** The primers used for qRT-PCR and plasmid construction.

## Data Availability

The entire *Ziziphus jujuba* Mill. genome sequence information was obtained from the Ensembl Genomes website (https://www.ncbi.nlm.nih.gov/assembly/GCF_000826755.1). *Arabidopsis thaliana* (Col-0) materials used in the experiment were supplied by Doctor. Xuan Zhao of Hebei Agricultural University, and this material was used with permission. The datasets supporting the conclusions of this study are included in the article and its additional files.

## Abbreviations

WT: *Arabidopsis thaliana* (Col-0)
OE: Overexpression *ZjRNase1*, *ZjRNase2*
qRT-PCR: Quantitative real-time PCR
MW: Molecular weight
PI: Theoretical isoelectric point
